# Receptor-Like Kinase LYK9 in *Pisum sativum* L. Is the CERK1-Like Receptor that Controls Both Plant Immunity and AM Symbiosis Development

**DOI:** 10.3390/ijms19010008

**Published:** 2017-12-21

**Authors:** Irina V. Leppyanen, Vlada Y. Shakhnazarova, Oksana Y. Shtark, Nadezhda A. Vishnevskaya, Igor A. Tikhonovich, Elena A. Dolgikh

**Affiliations:** All Russia Research Institute for Agricultural Microbiology, 196608, Podbelsky Shosse 3, St.-Petersburg, 196608 Pushkin, Russia; irina_leppyanen@mail.ru (I.V.L.); shahnazarova-v@mail.ru (V.Y.S.); oshtark@yandex.ru (O.Y.S.); olgastrunnikova@rambler.ru (N.A.V.); arriam2008@yandex.ru (I.A.T.)

**Keywords:** pea *Pisum sativum* L., Lysin-motif receptor-like kinase PsLYK9, AM symbiosis, phytopathogenic fungi, gene expression, knockdown regulation, *Agrobacterium rhizogenes*

## Abstract

Plants are able to discriminate and respond to structurally related chitooligosaccharide (CO) signals from pathogenic and symbiotic fungi. In model plants *Arabidopsis thaliana* and *Oryza sativa* LysM-receptor like kinases (LysM-RLK) AtCERK1 and OsCERK1 (chitin elicitor receptor kinase 1) were shown to be involved in response to CO signals. Based on phylogenetic analysis, the pea *Pisum sativum* L. LysM-RLK PsLYK9 was chosen as a possible candidate given its role on the CERK1-like receptor. The knockdown regulation of the *PsLyk9* gene by RNA interference led to increased susceptibility to fungal pathogen *Fusarium culmorum*. Transcript levels of *PsPAL2*, *PsPR10* defense-response genes were significantly reduced in *PsLyk9* RNAi roots. PsLYK9’s involvement in recognizing short-chain COs as most numerous signals of arbuscular mycorrhizal (AM) fungi, was also evaluated. In transgenic roots with *PsLyk9* knockdown treated with short-chain CO5, downregulation of AM symbiosis marker genes (*PsDELLA3*, *PsNSP2*, *PsDWARF27*) was observed. These results clearly indicate that PsLYK9 appears to be involved in the perception of COs and subsequent signal transduction in pea roots. It allows us to conclude that PsLYK9 is the most likely CERK1-like receptor in pea to be involved in the control of plant immunity and AM symbiosis formation.

## 1. Introduction

The ability of plants to distinguish between pathogenic and beneficial microorganisms determines their ability to successfully exist in soil. Plants possess a well-developed system to recognize certain surface components and the compounds of microorganisms released into the medium, which are called microbe-associated molecular patterns (MAMPs); these are perceived by plant pattern recognition receptors [1,2,3]. The recognition of a microorganism as a pathogen during early interactions triggers activation of the immune responses that prevent microbial penetration into plant cells. Plants are also able to develop symbiotic relationships with beneficial microorganisms like arbuscular mycorrhizal (AM) fungi and nitrogen-fixing bacteria belonging to the order *Rhizobiales*, known as rhizobia [4,5]. The development of symbiotic interactions is based on the ability of microorganisms to avoid the activation of the plant’s immune system or to suppress it for intracellular infection progression. To establish different types of plant–microbial relationships, microorganisms enter into a molecular dialog with plants.

Chitin and its derivatives, chitooligosaccharides (COs), with a high degree of polymerization, are typical MAMPs that are perceived by plants [6,7,8,9,10]. Long-chain COs produced by pathogenic fungi and invertebrates are detected as elicitors of defense reactions in plants that prevent microbial infection. In contrast, short-chain COs with degrees of polymerization 4 and 5 (CO4 and CO5) and lipochitooligosaccharides (LCOs), Myc factors and Nod factors, have been identified as the key signals of AM fungi and rhizobia, which enable entry of the microbes into plant root cells. Legume plants may be considered as unique objectives to study the pathogenic and mutualistic relationships of plants, as they are able to perceive all four types of chitooligosaccharidic molecular signals coming from microorganisms, but inducing various responses.

Receptors with LysM motifs in extracellular domains were shown to be required in the recognition of both bacterial and fungal LCOs and COs in plants [7,8,11,12,13,14,15,16,17,18,19,20,21,22]. In model plants *Arabidopsis thaliana* and *Oryza sativa*, Lysin-motif receptor-like kinases (LysM-RLKs) AtCERK1 and OsCERK1 (chitin elicitor receptor kinase 1) were shown to be essential for the recognition of chitin and long-chain COs with degree of polymerization 8 (CO8). It was found that in *A. thaliana* and *O. sativa*, the receptors AtCERK1 and OsCERK1 form homooligomeric AtCERK1/AtCERK1 or heterooligomeric complexes with additional receptor proteins (like AtLYK5/AtCERK1 and OsCEBiP/OsCERK1), which are involved in the regulation of plant resistance to infection [9,17,19]. In addition, studies based on phenotypic analyses of the chitin receptor mutant (*cerk1*) in rice *O. sativa* that—in contrast to *Arabidopsis*—was able to form symbiosis with AM fungi, showed that OsCERK1 was also involved in the recognition of short-chain COs with degree of polymerization 4 and 5 (CO4–CO5) through complex formation with an unknown co-receptor [20,21]. It is known that AtCERK1 and OsCERK1 were also important for peptidoglycan recognition; this has a structure that is similar to that of chitin, and it comprises the main component of the bacterial cell wall. To recognize peptidoglycan, receptor proteins form complexes like AtCERK1/AtLYM1/AtLYM3 and OsCERK1/OsLYP4/OsLYP6 [17,23]. This formation establishes CERK1 as playing a key role in the recognition of signal molecules of both fungal and bacterial origin.

Based on the results of a series of studies, an evolutionary relationship between LysM-RLK CERK1 and LysM-RLKs, which are involved in Nod factor perception and symbiosis regulation between plants and rhizobial bacteria like NFR1 (Nod factor receptor 1) in *Lotus japonicus* and LYK3 (LysM-type receptor-like kinase 3) in *Medicago truncatula*, was suggested [15,21,24]. Evidence in support of this assumption primarily comes from experiments on the activation of symbiotic genes, not only by Nod factors, but also by chitin and CO8 in *L. japonicus* wild-type plants and mutants impaired in *nfr1* gene [24]. Similar results were obtained during the analysis of transcriptomes of *L. japonicus* roots treated with CO8 [25]. It points toward the participation of receptor to chitin/long-chain COs in induction of symbiotic genes. At the same time, the treatment of *nfr1* mutant plants with chitin or CO8 showed that their recognition related to specific receptor, and recognition occurred independently of Nod factor receptor. Consequently, there are specific receptors for chitin and oligomers of chitin, which remain unknown in legume plants.

To investigate the interplay between symbiotic and defense pathways in the legume plant pea *P. sativum*, we were interested in searching for receptors involved in controlling plant immunity and symbiosis development. In this study, we questioned whether the *PsLyk9* gene regulates plant immunity and AM symbiosis development in pea plants, and whether it may also regulate the expression of the AM symbiosis-related markers and defense-response genes in roots treated with COs differing in degree of polymerization. We found that the *PsLyk9* gene was upregulated in response to pathogenic fungi infection, and its enhanced expression was observed in its roots during mycorrhization. Data on *PsLyk9* RNA interference (RNAi) suggest that the *PsLyk9* gene may regulate plant defense resistance to phytopathogenic fungi and AM symbiosis development. This indicates that PsLYK9 may be a suitable candidate for a CERK1-like receptor in pea.

## 2. Results

### 2.1. Searching for a Candidate among Pea Lyk Genes

To identify CERK1-like receptor in pea, we searched for genes encoding LysM-RLKs in this legume; 26 and 19 LysM-RLKs genes have been found in model legumes like *Medicago truncatula* and *Lotus japonicus* [26,27,28], while only three *P. sativum* L. genes of this family have been annotated in databases to date—*PsSym10* and *PsSym37*, *PsK1* [11,29]. Previously, it was shown that the *AtCERK1* in *Arabidopsis* has a high homology to genes in *M. truncatula* and *L. japonicus*, which belong to LysM-I phylogenetic groups including homologs of *MtLYK3* (LysM-type receptor-like kinase 3) and *LjNFR1* (Nod factor receptor 1) genes in these legumes [26,27,30]. *OsCERK1* in rice also has a high homology with *LjNFR1*/*MtLY*K3 in *M. truncatula* and *L. japonicus* that belong to the same groups [21]. LysM-I phylogenetic groups include additional ten (*MtLYK1*–*10*) and seven (*LjLYS1*–*7*) LysM-RLK genes that demonstrate a high level of homology with *MtLYK3* and *LjNFR1*, correspondently [26,28,30]. Representatives of LysM-I phylogenetic groups have from 10 to 12 exons and encode LysM receptors with potentially functional kinases (subfamily of LysM receptor like kinases, LYKs).

**Figure 1:**
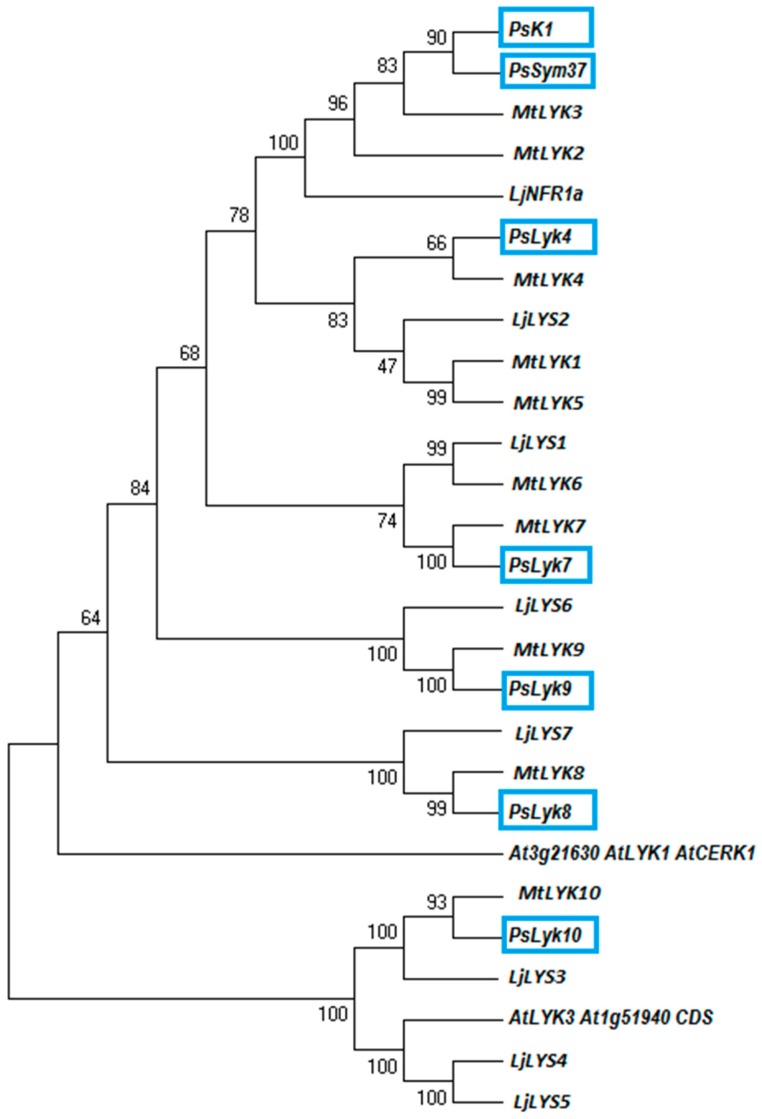
Phylogenetic tree analysis showing the evolutionary relationships of the legume representatives of the LYK group of LysM-RLKs. Maximum-likelihood branch lengths were calculated using full length encoding sequences of *LYK* genes. The blue boxes represent pea genes. The branches show the bootstrap values. Ps, *Pisum sativum*; Lj, *Lotus japonicus*; Gm, *Glycine max*; Mt, *Medicago truncatula*; At, *Arabidopsis thaliana*.

Based on the sequences of identified members of the *M. truncatula* and *L. japonicus* LysM-I phylogenetic groups, homologs were identified in the genome of *P. sativum* by searching in all available Transcriptome Shotgun Assemblies [31,32,33,34,35]. In silico searches for such LysM-RLKs resulted in identification of the full-length and partial transcripts for five additional predicted pea genes *PsLyk4*, *PsLyk7*, *PsLyk8*, *PsLyk9* and *PsLyk10* (Table S1; the names were given based on homology with *M. truncatula LYK* genes). Together with two other known *PsSym37* and *PsK1* genes (EU564096 and EU564088) from this phylogenetic group all these coding sequences were used for phylogenetic analysis (Figure 1). In addition, two *LYK* genes encoding receptor proteins with potentially functional kinases like *AtLYK1* (*AtCERK1*) and *AtLYK3* (At3g21630 and At1g51940) have also been used for analysis (Figure 1).

Pea *Lyk* genes demonstrated high level of homology with *M. truncatula* and *L. japonicus* genes (Figure 1). Among these genes, *PsSym37* and *PsK1* showed the highest level of homology with *MtLYK3* and *MtLYK2* as well as with *LjNFR1* encoding putative receptors to Nod factors in legumes [14,36]. *PsLyk10* demonstrated the highest level of homology with *MtLYK10* and *LjLYS3* that encode the legume receptor to rhizobial exopolysaccharides [37]. Based on the phylogenetic analysis, *AtCERK1* in *Arabidopsis* seems to show an evolutionary relationship with *MtLYK1/PsLyk1*–*MtLYK9/PsLyk9* genes, like it was previously shown for kinase domains of these receptor proteins (Figure 1) [26].

Among these genes, the *AtCERK1* (At3g21630) had the highest percent of identity 63.4% with *PsLyk9*, 61.9% with *PsSym37*, 61.4% with *PsK1*. Other pea *Lyk* genes showed a lower percent of homology with *AtCERK1*. Moreover, *PsLyk9* encoded the receptor protein with YAQ sequence in kinase domain [24] that makes it suitable candidate for the role of receptor involved in symbiosis development. We hypothesized that the *PsLyk9* gene may encode a CERK1-like receptor in pea.

**Figure 2:**
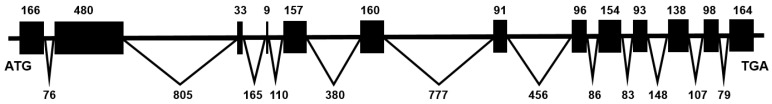
*PsLyk9* gene structure. The exon (boxes) and intron (line) size in nucleotides are presented in cv. Finale background.

To verify the structure of the *PsLyk9* gene in pea, we have cloned the full-length gene and its complementary DNA (cDNA) in cv. Finale using RACE analysis. Analysis showed that the *PsLyk9* gene in pea has 11 introns (Figure 2). The full-length cDNA of *PsLyk9* contained an open reading frame of 1845 nucleotides encoding a peptide of 614 amino acid residues with the predicated molecular mass of 67 kD.

### 2.2. Analysis of PsLYK9 Participation in Response to Exogenously Applied Elicitors and Infection with Phytopathogenic Fungi Fusarium Culmorum

**Figure 3:**
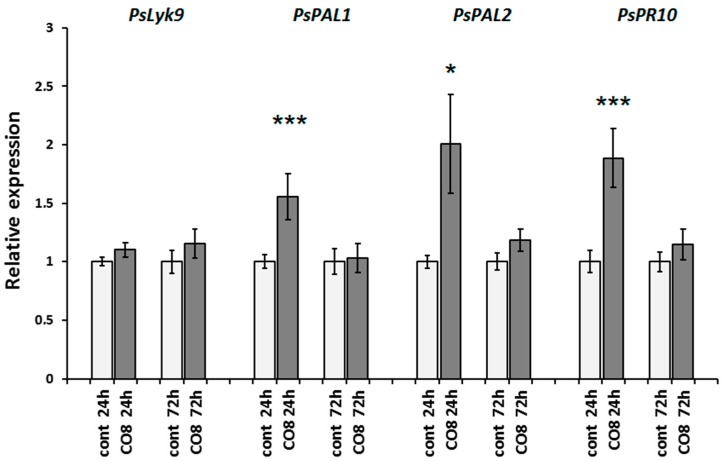
Transcript levels of the *PsLyk9* gene encoding LysM-RLK and *PsPAL1*, *PsPAL2*, *PsPR10* defense-response genes in pea roots treated with 10^−6^ M CO8 for 24 and 72 h. As a control, water-treated plants were used. The relative expression was normalized against the constitutively expressed ubiquitin and actin genes. Values are means ± SEM of three technical repeats. The graphs show the results of one biological repeat, representative for three biological independent experiments. Asterisks indicate statistically significant differences compared with the respective water-treated control: *** *p* < 0.001, * *p* < 0.05.

It was previously shown that pea plants can respond to chitosan oligomer treatment with a degree of polymerization of 7 or higher [38,39]. Indeed, our experiments revealed that pea plants treated with chitosan oligomers with a degree of polymerization of around 8 (CO8) respond best to the essential induction of *PsPAL1* and *PsPAL2* genes that encode various isoforms of the phenylalanine-ammonium-lyase (E.C.4.3.1.5) involved in phytoalexin synthesis in pea. It also induces the *PsPR10* gene (pathogenesis-related 10), which is used as a defense-response marker to elicitor treatment (Figure 3). The most significant response was detected 24 h after treatment, while at 72 h, the effect was reduced. Treatment with CO8 did not result in the significant induction of the *PsLyk9* gene, probably because the level of *PsLyk9* expression in untreated roots was high (Figure 3).

**Figure 4:**
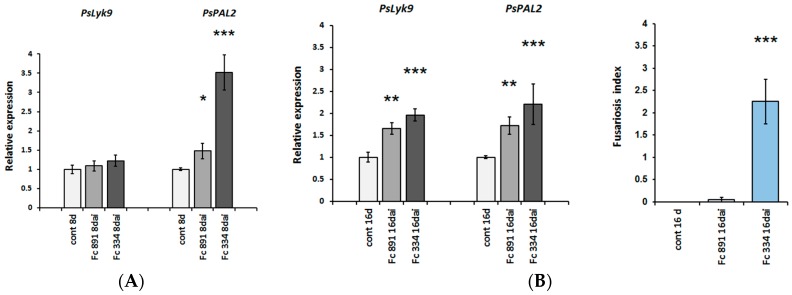
*PsLyk9*, *PsPAL2* gene expression in pea roots on days 8 (**A**) and 16 ((**B**), **left**) after infection with phytopathogenic fungi *F. culmorum*. Weakly pathogenic Fc 891 and highly pathogenic Fc 334 strains were used for infection. As a control, non-infected plants were used. Disease symptoms were evaluated in points on day 16 ((**B**), **right**), when the signs of disease began to appear on pea plants. No visible signs of disease were found on day 8 after infection. Asterisks indicate statistically significant differences compared with control non-infected plants: *** *p* < 0.001, ** *p* < 0.01, * *p* < 0.05.

To verify the influence of PsLYK9 on plant interactions with phytopathogenic fungi, two *F. culmorum* (Wm. G. Sm.) Sacc. 334 and 891 strains, which differ in their ability to cause symptoms of disease in pea plants, were applied [40]. On day 16 after infection, when the visible signs of disease began to appear on pea plants, both *F. culmorum* strains induced the expression of *PsLyk9* and *PsPAL2* in plant roots (Figure 4). The effect was the most pronounced for the highly aggressive strain 334, probably due to higher pathogenic load. Since the *PsLyk9* gene is induced in response to infection with both weakly and highly pathogenic fungal strains, it may indicate that the receptor kinase encoded by this gene participates in the development of non-specific immune response in plants toward infection with phytopathogenic fungi acting as a typical pattern recognition receptor. To further investigate it, the transcript level of the *PsLyk9* gene can be evaluated at earlier stages of pea—*F. culmorum* interaction.

### 2.3. Effect of PsLyk9 Gene Repression on the Transcription Level of Defense-Response Genes

**Figure 5:**
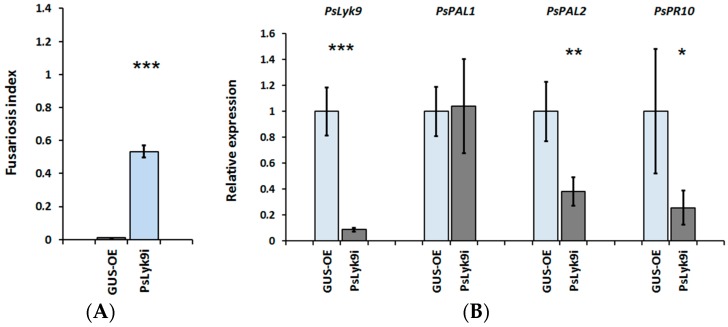
Effect of *PsLyk9* RNAi on the resistance of pea plants to the weakly pathogenic fungus *F. culmorum* 891 and the transcript levels of defense-response genes. (**A**) Disease level in *PsLyk9*-RNAi and GUS-OE transgenic pea plants; (**B**) The expression levels of the *PsPAL1* and *PsPAL2*, *PsPR10* defense-response genes in transgenic pea roots with *PsLyk9* knockdown; GUS-OE—transgenic pea plants transformed with pB7WG2D-GFP construct (control); *PsLyk9*-RNAi—transgenic pea plants transformed with the pK7GWIWG2D-*PsLyk9i* construct. Error bars indicate the SEM of seven-ten biological repeats. Asterisks indicate statistically significant differences compared with control water-treated roots: *** *p* < 0.001, ** *p* < 0.01, * *p* < 0.05.

To analyze the *PsLyk9* gene function in detail, an RNAi construct was generated (*PsLyk9i*) and introduced in pea plants, targeting *PsLyk9* mRNA (Figure 5). In *PsLyk9i* transgenic roots, the expression of the *PsLyk9* gene decreased significantly by 80% compared with control roots (GUS overexpressing control, GUS-OE). In transgenic roots with *PsLyk9* knockdown, we expected to see an increase in their sensitivity to the weakly pathogenic fungus strain *F. culmorum* 891. Indeed, it was found that in *PsLyk9i* pea roots, suppression of *PsLyk9* correlated with an increased sensitivity to the weakly pathogenic strain *F. culmorum* 891 in comparison with the control GUS-OE plants (Figure 5A). Signs of *Fusarium* damage were observed in all examined roots, while no evidence of disease was detected in control plants. Quantitative reverse transcription polymerase chain reaction (qRT-PCR) analyses of RNA extracted from individual transgenic roots (*n* = 7–10) demonstrated that roots carrying the *PsLyk9-*RNAi construct displayed reduced transcription levels in *PsPAL2* and *PsPR10* gene expression (Figure 5B).

In addition, *PsLyk9* RNAi composite plants were transferred in glass jars containing CO8 and exposed for 24 h. The analysis showed decreased expression levels of *PsPAL2* and *PsPR10* in *PsLyk9* RNAi roots in response to exogenously applied CO8 compared with GUS-control plants. Thus, we have shown that the receptor kinase PsLYK9 can have a direct effect on plants’ resistance to infection with respect to phytopathogenic fungi and the expression of defense-response genes during plant treatment with CO8.

### 2.4. Analysis of Expression Levels of the Genes Involved in Developing Arbuscular–Mycorrhizal Symbiosis in Wild-Type Pea Plants and Transgenic Plants with PsLyk9 Gene Repression

To test the hypothesis that the PsLYK9 receptor is involved not only in the control of plant resistance to infection with phytopathogenic fungi, but also in the regulation of symbiosis development with AM fungi, the analysis of marker gene expression levels, activated in response to AM fungi inoculation, was performed. Previously, the search for such markers was performed in pea plans. Three genes—*PsAM1*, *PsAM3* and *PsAM5* (PSU43401, AJ006305, AJ276507)—were shown to be induced at the initial stages of the plant’s interaction with AM fungi, corresponding to the presymbiotic phase and early penetration [41,42]. Subtractive hybridization and macro-array analysis enabled the identification of a set of pea mycorrhiza-regulated genes like *PsMAPK* (AJ308148) and *PsDRR 232a* (AF139018) [43]. We also found homologs of well-known *M. truncatula* AM symbiosis markers like *MtGST* (Medtr5g076900), *MtPT4* (Medtr1g028600), *Myb-TF* (Medtr7g068600), *MtRAM1* (Medtr7g027190), and *MtRAM2* (Medtr1g040500) in pea [44,45,46,47,48,49,50,51]. In addition, an analysis of *PsNSP1* (KT454934), *PsNSP2* (EU736106), *PsDWARF27* (GDTM01031662), and *PsDELLA1*–*3* (DQ848351, DQ845340, GDTM01011926) gene expression was performed in pea plants, because it was found that they were important in AM symbiosis development in pea and other legumes [52,53,54,55,56,57,58].

Finally, the expression level of 16 genes was tested in the roots of cv. Finale inoculated with AM fungus, *Rhizophagus irregularis*, at 14 and 28 days after inoculation. At both terms, all analyzed pea plants were extensively colonized by the fungus. The intensity of internal colonization of the root system (M%) was on an average 17.5% and 35.0%, respectively, and the arbuscule abundance in mycorrhizal root fragments (a%) was 29.4% and 36.5%, respectively.

**Figure 6:**
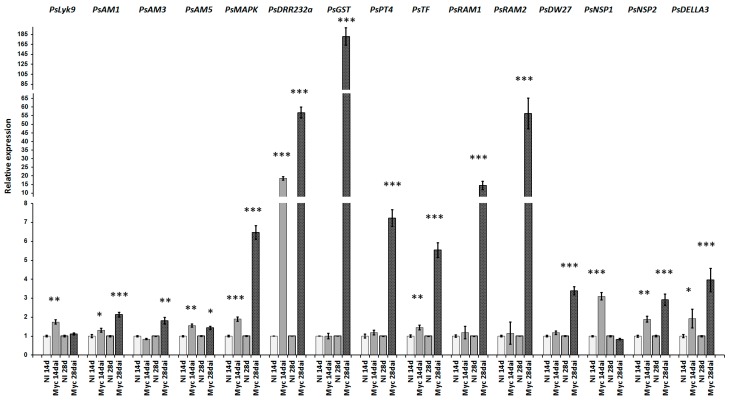
Up-regulation of the AM symbiosis-related marker genes in pea roots on 14 and 28 days after inoculation with *R. irregularis*. The relative expression was normalized against the constitutively expressed ubiquitin and actin genes. Results are means ± SEM of three technical repeats. The graphs show the results of one biological repeat, representative for three independent experiments. Asterisks indicate statistically significant differences compared with control water-treated roots: *** *p* < 0.001, ** *p* < 0.01, * *p* < 0.05.

The analysis showed an increase in the expression of 13 genes (*PsAM1*, *PsAM3*, *PsMAPK*, *PsDRR232a*, *PsGST*, *PsPT4*, *PsMyb-TF*, *PsRAM1*, *PsRAM2*, *PsNSP1*, *PsNSP2*, *PsDWARF27*, and *PsDELLA3*), that may be considered as markers of AM symbiosis development in pea (Figure 6).

**Figure 7:**
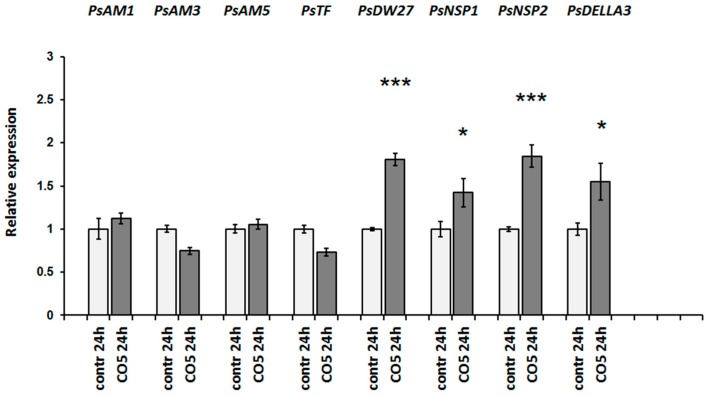
Quantitative reverse transcription polymerase chain reaction (qRT-PCR) analysis of transcription levels of the AM symbiosis-related marker genes in pea roots treated with 10^−5^ M CO5 for 24 h. As a control mock-treated plants were used. The relative expression was normalized against the constitutively expressed ubiquitin and actin genes. Values are means ± SEM of three technical repeats. The graphs show the results of one biological repeat, representative for three biological independent experiments. Asterisks indicate statistically significant differences compared with the respective mock-treated control: *** *p* < 0.001, * *p* < 0.05.

Since the changes in gene expression during mycorrhiza inoculation occur in response to the combined effect of both short COs and Myc factors, we also tested the activation of these markers in response to exogenously applied chitin oligosaccharide CO5. In the case of CO5, concentrations of 10^−6^ and 10^−5^ M were used to estimate the effect of these signal molecules on plants. All CO5 treatments were carried out for 24 h. Both CO5 concentrations were active in the induction of responses, but the effect of 10^−5^ M was more pronounced. The activation of at least four genes (*PsDELLA3*, *PsNSP1*, *PsNSP2*, and *PsDWARF27*) after treatment with CO5 was found in our experiments (Figure 7).

**Figure 8:**
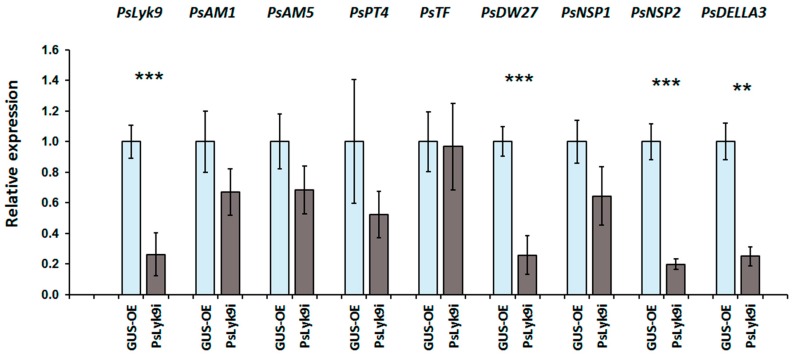
Reduction in gene expression level of *PsDWARF27*, *PsNSP1*, *PsNSP2*, and *PsDELLA3* in the transgenic pea roots with *PsLyk9* knockdown treated with 10^−5^ M CO5 for 24 h. GUS-OE—transgenic pea plants transformed with pB7WG2D-GUS construct (control); *PsLyk9i*—transgenic pea plants transformed with the pK7GWIWG2D-*PsLyk9i* construct. Error bars indicate the SEM of seven-ten biological repeats. Asterisks indicate statistically significant differences compared with control (GUS-overexpressing plants (GUS-OE)): *** *p* < 0.001, ** *p* < 0.01.

At the next stage of our study, we analyzed the expression levels of AM symbiosis development marker genes in the *PsLyk9* RNAi transgenic roots in response to CO5 treatment (Figure 8). It was shown that in response to treating the *PsLyk9* RNAi roots with CO5, there was no activation of the expression of four markers (Figure 8), although the decrease in *PsNSP1* transcription was not statistically significant due to its high variability. Thus, this finding suggests that the receptor kinase PsLYK9 participates in the development of AM symbiosis in pea and controls plants’ response to recognizing COs with a low degree of polymerization.

Activation of *PsNSP1*, *PsNSP2*, and *PsDWARF27* may be related to strigolactone (SL) biosynthesis in plants during AM symbiosis development [59]. Therefore, the regulation of SL biosynthesis by *NSP1* and *NSP2* may be an ancestral function conserved in higher plants.

### 2.5. Analysis of Transcription Levels of the Cytokinin Response Genes in Pea Roots

**Figure 9:**
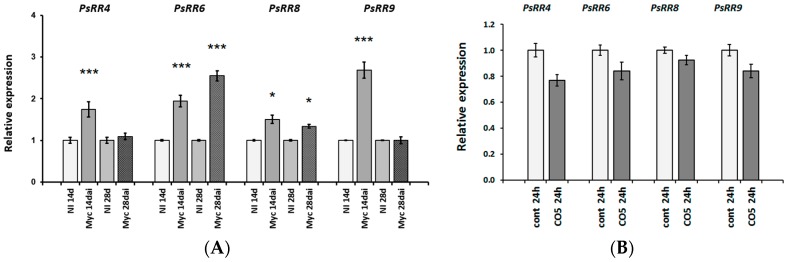
Relative expression of type-A cytokinin response regulator genes *PsRR4*, *PsRR6*, *PsRR8*, *PsRR9* was determined using qRT-PCR. (**A**) Analysis of pea roots on 14 and 28 days after inoculation with *Rhizophagus irregularis* (dai). NI, not inoculated; (**B**) Effect was estimated in pea roots treated with 10^−5^ M CO5 for 24 h. As a control mock-treated roots were used. The relative expression was normalized against the constitutively expressed ubiquitin and actin genes. Asterisks indicate statistically significant differences compared with control (not inoculated or not treated plants): *** *p* < 0.001, * *p* < 0.05.

It is known that hormones, particularly cytokinins, may participate in the formation of AM symbiosis in plants. Increased levels of trans-zeatin riboside were found in the mycorrhizal roots when compared with non-mycorrhizal roots in some legume plants [60]. The activation of type-A and type-B cytokinin response regulators (type-A *RR* and type-B *RR*) was shown to be related to cytokinin receptor activation in legume plants [61,62,63]. Indeed, our experiments showed that the expression of type-A cytokinin response genes *RR4*, *RR6*, *RR8*, and *RR9* increased in wild-type roots within 14 days of infection with AM fungi (Figure 9A).

To verify whether these cytokinin response genes can be activated by CO5 during the development of AM symbiosis, we estimated the level of A-type *RR4*, *RR6*, *RR8*, and *RR9* expression after treatment with signal molecules. However, we were not able to see any differences in the expression of these genes in treated and control pea roots (Figure 9B). Therefore, during mycorrhization, the induction of cytokinin response genes was probably related to Myc factors, not to CO4–CO5 molecules.

## 3. Discussion

Here, we report the characterization of the pea LysM-RLK PsLYK9 involved in recognizing COs with various degrees of polymerization, and which is implicated in the regulation of both plant resistance to phytopathogenic fungi and the control of AM symbiosis development.

In plants, LysM-RLKs have been involved in the recognition of chitin and COs from fungi, bacterial peptidoglycans, and various LCOs like Myc factors and Nod factors, which are known to be symbiotic signals produced by rhizobia and AM fungi [8,9,12,13,14,15,17,23]. Among them, the LysM-RLKs AtCERK1 and OsCERK1 can recognize fungal chitin/COs and bacterial peptidoglycans [8,9]. They act as typical pattern-recognition receptors that activate pathogen-triggered immunity pathways and confer resistance through the activation of defense responses to limit pathogen growth. Recent studies have also shown that OsCERK1 may fulfill dual functions and involved in recognizing structurally similar signals not only from phytopathogenic fungi (like long-chain CO8), but also from symbiotic AM fungi (like short-chain CO4 and CO5) [20,21]. We suggested that in pea plants that can form symbiotic relationships with AM fungi, a CERK1-like receptor may also fulfill a dual function.

Based on the phylogenetic analysis, we determined that the pea LysM-RLK LYK9 may be a possible candidate as a CERK1-like receptor. The knockdown of the *PsLyk9* gene by RNA interference (*PsLyk9*-RNAi) resulted in the down-regulation of *PsPAL2* and *PsPR10* defense-response genes in roots, and this led to greater susceptibility to fungal pathogens *F. culmorum* producing a mixture of COs with various degrees of polymerization. In addition, the responses to exogenously applied CO8 elicitors were reduced in *PsLyk9* RNAi plants. These results clearly indicate that PsLYK9 appears to be involved in the perception of CO elicitors and subsequent signal transduction in pea roots.

Since legume plants can form symbiotic relationships with AM fungi, we also evaluated PsLYK9 involvement in the recognition of short CO5 as primary signals of AM fungi. The knockdown of *PsLyk9* greatly affected the gene responses induced by short COs (i.e., CO5), the most numerous signals of AM fungi. The results of this study further indicate that PsLYK9 may also be involved in the recognition of CO5 in pea roots.

While preparing this manuscript, a paper by Bozsoki et al. [64] was published that described similar results; the team’s investigation also showed the critical role played by LysM-RLK LjLYS6/MtLYK9 when recognizing chitin-based signal molecules in *L. japonicus* and *M. truncatula*. The screening of mutant collections enabled the identification of those mutants impaired in *lys6* and *lyk9* genes, which were defective in triggering chitin/COs-based responses in these legume plants. Therefore, our results were consistent with findings of Bozsoki et al. [64] in *L. japonicus* and *M. truncatula*. It seems like the pea *PsLyk9* gene is a likely ortholog of the *LjLYS6* and *MtLYK9* genes in two model legumes.

One of the aims of our research was to identify those genes that are specifically involved in signal transduction in response to CO5 recognition. Studies of model legumes showed that components of a common signal pathway, like *MtDMI2*/*LjSYMRK*, *MtDMI1*/*LjPOLLUX*, *MtDMI3*/*LjCCaMK*, *MtIPD3*/*LjCYCLOPS*, *MtNSP1*/*LjNSP1*, and *MtNSP2*/*LjNSP2* were involved in triggering signal transduction via signal molecules, the Myc factors from AM fungi [53,55,65,66]. Myc factors trigger calcium spiking, lateral root formation and a definite set of genes in legume plants [53,67]. An analysis of mutants showed that the MtDMI2/LjSYMRK and MtDMI1/LjPOLLUX regulators were also necessary for triggering signal transduction by short CO4 and CO5, which can induce calcium spiking and a specific gene expression [25,68]. Here, we demonstrated that exogenously applied CO5 upregulated *PsDELLA3*, *PsNSP1*, and *PsNSP2* in pea roots. This may suggest that DELLA3, NSP1, and NSP2 are also involved in signal transduction activated by short COs. Although the transcriptome profiling of *M. truncatula* and *L. japonicus* roots revealed upregulation of the *NSP1* and *NSP2* genes encoding TFs by Myc factors [67], but not CO5 10^−8^ М [25], we suppose it may be connected to higher concentrations of applied CO5 in our experiments. To further investigate whether the PsDELLA3, PsNSP1, and PsNSP2 are involved in the CO4–CO5-activated signaling pathway, the effect of exogenously applied CO5 on *della*, *nsp1* and *nsp2* mutants should be estimated.

Since DELLA proteins are able to stimulate NSP1/NSP2 complex formation [58], a model may be proposed for a CO5-activated signal pathway in which DELLA3 and NSP1/NSP2 function as symbiotic regulators to directly activate the expression of DWARF27. DWARF27 is required for strigolactone biosynthesis; thus, cross-talk between gibberellin and strigolactone signaling during AM symbiosis may be mediated through DELLA3, NSP1, and NSP2. Conversely, no changes in the transcription level of the *RAM1* and *RAM2* genes, which are involved in the regulation of cutin synthesis during AM symbiosis [49], were found in our experiments. This may suggest that Myc factors, and not CO4–CO5, are involved in the activation of this pathway.

Recent studies showed that ERN and RAD1 transcription factors may play an important role in AM symbiosis development [45,69,70]. Therefore, whether CO5 can stimulate the expression of other transcription factors like ERN1, ERN2, and RAD1, which are involved in the regulation of various stages of AM symbiosis development, remains to be elucidated.

It was previously shown that trans-zeatin riboside accumulates to higher levels in mycorrhizal when compared with non-mycorrhizal roots [60]. This suggests that the cross-talk between plant hormones, cytokinins, and LCO or CO signaling may take place in AM symbiosis. It was interesting for us to determine that CO5 may induce the expression of cytokinin response genes in the roots of pea plants. However, the absence of cytokinin response gene activation in our experiment suggests that the function of CO signals is probably restricted to the presymbiotic AM stages, and that they do not activate genes related to cytokinin activation.

## 4. Materials and Methods

### 4.1. Plant Material and Germination Conditions

*Pisum sativum* L. cv. Finale seeds were surface sterilized for 5 min in concentrated sulfuric acid, then washed 3 times with sterile water, transferred to Petri dishes with 1% water agar and germinated in the dark at room temperature for 4–5 days. After germination (for 4–5 days), plants were transferred into pots with vermiculite or mineral substrate and grown in a growth chamber at 21 °C in a 16 h/8 h light/dark cycle at 60% humidity.

Seeds of chives *Allium schoenoprasum* L. were surface-disinfected as follows: 1 min in 96% ethanol, a rinse with sterile water, 5 min in 0.15% potassium permanganate aqueous solution, and a thorough rinse with sterile water. They were germinated on sterile humid filter paper in Petri dishes for 3–5 days at 27 °C in the dark and for 1–2 days at room temperature in the light. The seedlings were planted into pots (15 per pot) containing substrate with *Rhizophagus irregularis* inoculum. After 4–6 weeks, chive plants with mycorrhized roots were transferred to individual pots (1 per pot) and grown for 2–4 weeks.

### 4.2. Treatment of Pea Seedlings

Firstly, 4–5 days old pea seedlings were transferred in glass jars with 10^−6^–10^−5^ M water solutions of CO5 or CO8 and incubated for 24 h. Fully acetylated COs with degree of polymerization five (CO5) (Megazyme) and deacetylated COs with main degree of polymerization around eight (CO8) Mn = 1089, Mw = 1514, Ip = 1.39, CDS = 93% in Cl-form (The Center of Bioengineering Russian Academy of Science) were used for treatment. After treatment, the pea root fragments corresponding to responsive zones were harvested for RNA extraction and gene expression analysis.

### 4.3. Fusarium Culmorum Infection

Two strains of phytopathogenic fungi *Fusarium culmorum* (Wm. G. Sm.) Sacc. (weakly pathogenic 891 and highly pathogenic 334 strains) were used to infect pea plants. To obtain inoculum, *F. culmorum* 891 or 334 strains were grown on Chapek’s agar for 10–14 days and then washed from the plates with sterile water. 4–5 day old pea seedlings cv. Finale or *PsLyk9i* transgenic pea plants were transferred in pots with vermiculite saturated with Jensen medium [71], containing 3 × 10^5^ conidia of the phytopathogenic fungus *F. culmorum* 891 or 334 strains. The control plants were grown in sterile vermiculite saturated with Jensen medium. Infected root tissue was harvested at different stages after inoculation together with the uninoculated control roots.

### 4.4. Rhizophagus Irregularis Infection

The isolate of the symbiotic fungus *Rhizophagus irregularis* BEG144 was provided by the International Bank of Glomeromycota (Dijon, France) as a soil-root based inoculum from onion (*Allium cepa* L.) pot cultures. The isolate was used to produce the chives *A. schoenoprasum* L. nurse culture to infect wild type pea plants with mycorrhizal fungus (see Section 4.1).

Three pea seedlings were planted into each nurse pot around a mycorrhizal chive plant (3 pots per variant). All plants were grown in 300 mL ceramic pots, with a mineral substrate, the silica rich marl, supplemented with 1 g/L calcium orthophosphate. Pots with substrate were sterilized by autoclaving for 60 min at 134 °C and 0.22 MPa. Plants were grown in a growth room under the following conditions: 16/8 h day/night, 21 °C, relative humidity 60% and were fed once a week with modified Hoagland’s solution without phosphate (50 mL per pot) [57,72], and watered as needed. The expression of AM symbiosis specific genes was assessed on 14 and 28 days after infection. AM development was quantitatively assessed as described earlier [57].

### 4.5. Molecular Cloning

To obtain a genetic construct for downregulation of the *PsLyk9* gene, a fragment of 281 b.p. was amplified using cDNA cv. Finale as a matrix with primers flanked by the attB sites (Table S2) and cloned in the pDONR221 vector using BP clonase (Thermo Fisher Scientific, Waltham, MA, USA). Finally, the fragment was cloned in the pK7GWIWG2D vector using LR clonase enzyme. pK7GWIWG2D vector used in this work contained the green fluorescent protein (GFP) marker gene for the selection of transgenic roots. The resulting vector (*PsLyk9*-RNAi) contained a hairpin construct flanked by the 35S promoter and terminator sequences. The primers used for cloning are listed in Table S2. The verified construct was transferred into *Agrobacterium rhizogenes* strain Arqua 1 using electroporation method.

### 4.6. Agrobacterium Rhizogenes-Mediated Plant Transformation

For pea transformation, the seeds were sterilized as described above. Seeds were germinated on 1% water agar in the darkness. Then, 4–5 days old seedlings were transferred in sterile dark plastic bags with Jensen medium to the light and incubated for 2–3 days. The pea roots were cut off in the area of the hypocotyl and transformed with freshly grown *A. rhizogenes* strain Arqua 1. Plants were put in plastic jars (Duchefa Biochemie, Haarlem, The Netherlands) on Jensen agar, and the area of cut-off was covered with wet wool and foil. The seedlings were co-cultivated with *A. rhizogenes* for 10–14 days at 21 °C (16 h/8 h light/darkness) and subsequently transferred to Emergence medium [73] containing 150 mg/mL of cefotaxime. Plants were incubated on Emergence medium with antibiotic for 5 days and then transferred to fresh Emergence medium without antibiotic and additionally incubated for 3–5 days until the new roots were formed. Emerging roots were analyzed using an epifluorescent stereomicroscope (Stereo Discovery V8; Carl Zeiss, Göttingen, Germany). To analyze the effect of COs treatment, the plants were placed into glass jars and incubated with CO5 for 24 h.

### 4.7. RNA Isolation and cDNA Synthesis

Pea roots were harvested and frozen in liquid nitrogen. Total RNA was isolated using about 50–100 mg tissue per sample. Samples were thoroughly ground in a mortar to a fine powder in liquid nitrogen, at least three biological replicate per each condition. RNA was extracted using TriZol reagent (Thermo Fisher Scientific). After a DNase (Thermo Fisher Scientific) treatment, the samples were extracted with an equal volume of chloroform, and RNA was precipitated from the aqueous phase with 3 M sodium acetate and ethanol and subsequently quantified with a spectrophotometer UV-1280 (Shimadsu, Kyoto, Japan). RNA purity was checked by measuring spectrophotometric ratios of A260/A280 nm about 2. The efficacy of the DNase treatment was checked by using controls without reverse transcriptase for subsequent quantitative reverse transcription PCR (qPCR) analysis. RNA (from 1 to 2.5 μg) was used for cDNA synthesis with RevertAid Reverse Transcriptase (Thermo Fisher Scientific) for 1 h at 42 °C followed by heating to 70 °C, 10 min. Aliquots of the cDNA were diluted 1:10 for qPCR analysis.

### 4.8. Quantitative Reverse Transcription Polymerase Chain Reaction (qRT-PCR) Analysis

The qRT-PCR analysis was performed on a CFX-96 real-time PCR detection system with a C1000 thermal cycler (Bio-Rad Laboratories, Hercules, CA, USA), and SYBR Green intercalating dye were used for detection (Bio-Rad Laboratories). Each PCR reaction was carried out in a total volume of 10 µL. The following PCR program was used: 95 °C for 30 s, 40 cycles of 95 °C for 30 s, 54 °C for 30 s, 72 °C for 30 s. All reactions were done in triplicate and averaged. Cycle threshold (*C*t) values were obtained with the accompanying software and data were analyzed with the 2^–ΔΔ*C*t^ method [74]. The relative expression was normalized against the constitutively expressed ubiquitin and actin genes in pea. All the primer sets used in the expression analysis are listed in Table S2. All primer pairs were designed using Vector NTI program and produced by Evrogen Company (Moscow, Russia) (www.evrogen.com). Each experiment was repeated at least three times with independent biological samples.

### 4.9. Statistical Methods and Computer Software

The expression levels of the gene of interest (GOI) relative to the reference genes Ubiquitin and Actin were calculated for each cDNA sample using the CFX Manager™ software version 2.1 (BioRad Laboratories, Richmond, CA, USA). The expression levels of GOI were calculated as ratio of treated samples to control samples. Statistical analysis was conducted by SIGMAPLOT 13.

Multiple alignment of nucleotide sequences was performed using Clustal W [75] using Vector NTI Advance 10 (InforMax, http://www.informaxinc.com). MEGA6 was used to generate graphic output of phylogenetic tree [76]. One-way ANOVA and Student’s *t*-test were used to compare gene expression levels in transgenic roots.

## 5. Conclusions

We identified a LysM receptor-like kinase (LysM-RLK) PsLYK9 required for CO recognition and implicated in the regulation of both plant immune responses and the control of AM symbiosis development.

## Supplementary Materials

Supplementary materials can be found at www.mdpi.com/1422-0067/19/1/8/s1.
