# Supplementary material for: Receptor-Like Kinase LYK9 in Pisum sativum L. Is the CERK1-Like Receptor that Controls Both Plant Immunity and AM Symbiosis Development
Source: Int J Mol Sci. 2017 Dec 21;19(1):8. doi: 10.3390/ijms19010008 (PMC5795960; doi:10.3390/ijms19010008)
Supplement: Supplementary file 1 [file ijms-19-00008-s001.pdf]

Table S1. BLAST searches in different pea transcriptome databases of the *P. sativum Lyk* genes.

Medicago truncatula Mt4.0v1	*Pisum sativum* cv. SGE http://blast.ncbi.nlm.nih.gov/ (Zhukov et al., 2015)	*Pisum sativum* cv. Cameor http://bios.dijon.inra.fr/	*Pisum sativum* http://blast.ncbi.nlm.nih.gov/ (other projects)	*Pisum sativum* GenBank accession number
MtLYK1 AY372401, (Medtr5g086540) XM_003616943	-	-	-	-
MtLYK2 BN001116, (Medtr5g086310, Medtr5g086330) XM_003616924 XM_003616926	-<br>-	-<br>-	GAMJ01007644<br>GEUU01084920	*PsK1* EU564096,<br>*PsSym37* EU564088
MtLYK3 AY372406, (Medtr5g086130) XM_003616910	-<br>-	-	GAMJ01007644<br>GEUU01084920	*PsK1* EU564096,<br>*PsSym37* EU564088
MtLYK4 AY372407, (Medtr5g086120) XM_003616909	GDTM01035166	PsCam010968	GCMK01002635 GCMI01007867	-
MtLYK5 BN001117, (Medtr5g086090) XM_003616906	-	-	-	-
MtLYK7 AY372405, (Medtr5g086030) XM_003616900	GDTM01003580	-	GEZC01028741 GEZC01034730 GCMF01025080	-
MtLYK8 (Medtr2g024290) XM_013607437	GDTM01039384	-	GCMO01034719 GEUU01078867	-
MtLYK9 (Medtr3g080050) XM_003601328	GDTM01035167 GDTL01025510	-	GEZC01062852	-
MtLYK10 (Medtr5g033490) XM_003613117	GDTM01015333	PsCam036987	GCMK01009418	-

Table S2. List of primers.

Gene	Product	Primers
PsLyk9	LysM-receptor-like kinase	F - TCCACTATGCTAATTTAACCAATGTTC<br>R - TCGCAAGTATCCAAAATATACACCAG
PR1	Disease resistance protein	F - GGGGTCCATATGGTGAGAAC<br>R - TAATAACCAGGTGGATCATAGTTACA
PR10	Disease resistance protein PR10-1 (DRR49a), Putative RNAse	F - GCCGGAACCATCAAGAAACT<br>R - GCCTTGAAAAGACCATCACCC
PAL1	Phenylalanine ammonium lyase 1	F - TGAGAATCAACACACTTCTCCAAGG<br>R - GCATTAAGAATTTCCCCAGAGGTC
PAL2	Phenylalanine ammonium lyase 2	F - TGAGAATCAACACACTTCTCCAAGG<br>R - AGCATTAAGTATTTCTCCAGACGGTC
AM1	Putative peroxidase	F - TGCTAGCTAGCACTGCTCTACTA<br>R - GCTAGCGAGTACGCTACGGG
AM3	Unknown protein	F - TGCTAGCTACGATCGACTCGTGCC<br>R - TAGCTAATCTTAGCGCGTTATGC
AM5	Putative proteinase	F - GTATTCGGGCTATTGTATTCGCGT<br>R - ATCTCGCGTATTTGCGCGATTTC
DRR 232a	Disease resistance protein DRR 232a	F - CGTATTACGGGCTATAGCGGTCT<br>R - GCTATTAGCGGCAATTATCTGGTGT
GST	Glutathione-S-transferase	F - CGATCTTATCGGTCGCATGTCTGT<br>R - GCTACATCGGGTATTCGTCGATTC
PT4	Inorganic phosphate transporter 4	F - CATTCTGAGCTGATCGTCGTATGGTG<br>R - GCATTAAGAATTTCCCCAGAGGTC
DWARF27	Iron-containing protein required for the biosynthesis of strigolactones	F - GTATTATTATGCTCGCCCCTAGCTAC<br>R - TATTATATCGGCGGTACACTGCAC
DELLA	DWARF8-like GAI protein	F - TCGTAGCGACATCCTCTCTCTACTAGG<br>R - TGCATGCCTGGCCTATTTATACGCTTCG
NSP1	GRAS family transcription factor	F - TATTGCTACGTCTAGTAGTCTTAGCTG<br>R - GCTACTAGCGTCTCATCTAGCTAACC
NSP2	GRAS family transcription factor	F - TACGTACTCCTGGGTAGCTGCTGTAC<br>R - TCGTACGTCGTACGTACGTAGCTGA
RAM1	GRAS family	F - TTAGCTGTACGTACGTACGTACGTAGTC

	transcription factor	R - TCGTACGCCTTCTGAGTCTCTAGGGTC
Myb-TF	Myb- transcription factor	F - GCTACGTGGCGCTATATATCGGGC<br>R - TGGCCTATCGTACTCCGTTTAGG
RAM2	Glycerol-3-phosphate acyltransferase	F - GCTACTATTAAATTCTCCCTTCGCCG<br>R - CTAGCTGTAGCTATATACCTACTTG
